# Multiple cardiac myxofibrosarcomas with complete right pulmonary artery occlusion: a case report

**DOI:** 10.3389/fonc.2023.1197463

**Published:** 2023-09-04

**Authors:** Weimin Zhang, Qi Tang, Balhen Bolathan, Yan Xing, Xiaoxin Sun, Qiang Huo

**Affiliations:** ^1^Department of Cardiac Surgery, The First Affiliated Hospital of Xinjiang Medical University, Urumqi, Xinjiang, China; ^2^Cardiac Ultrasound Department, The First Affiliated Hospital of Xinjiang Medical University, Urumqi, Xinjiang, China; ^3^Imaging Center, The First Affiliated Hospital of Xinjiang Medical University, Urumqi, Xinjiang, China; ^4^Nuclear Medicine Department, Fuwai Hospital, Chinese Academy of Medical Sciences, Beijing, China

**Keywords:** cardiac tumor, myxofibrosarcoma, primary, complete right pulmonary artery occlusion, surgery

## Abstract

Primary cardiac myxofibrosarcoma is a rare form of cardiac malignant tumors. MFS usually involves the left atrium and presents as a unicentric or multicentric tumor mass. We reported on a 37-year-old female who presented with chest tightness and dyspnea for a month, dry cough, and occasionally having blood streak sputum for half a month. Echocardiography, cardiac computed tomography and cardiac positron emission tomography revealed multiple tumors in the heart. The right ventricle and right pulmonary artery were involved, with occlusion of the right pulmonary artery. Cardiac tumors were surgically resected and were consistent with low-grade MFS. No recurrence or metastasis occurred at 20 months of follow-up.

## 1 Introduction

Primary cardiac tumors are rare, with an incidence of 1.38–30 per 100,000 per year (1). They are usually benign, and only 20% are malignant (2). Primary cardiac sarcoma is a rare clinical entity, with an incidence of 0.0001% in collected autopsy series (3). However, myxofibrosarcoma (MFS) is an uncommon primary cardiac sarcoma that typically affects the left atrium (LA). Only a few isolated cases of primary cardiac MFS have been reported (4). Herein, we describe a clinicopathologic case of primary MFS in the right ventricular outflow tract (RVOT) and right pulmonary artery (RPA), with occlusion of the RPA.

## 2 Case report

**Figure 1:**
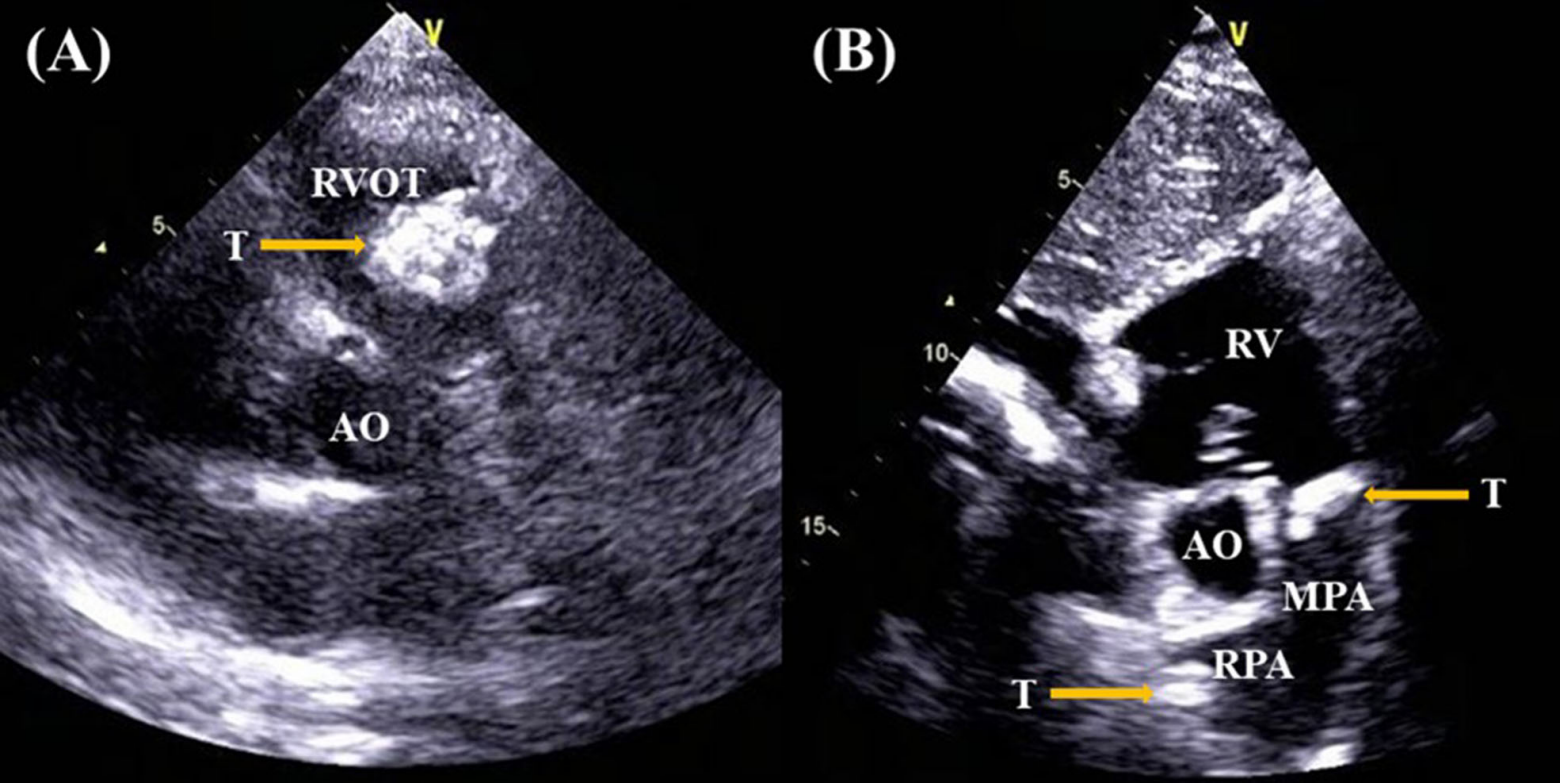
Transthoracic echocardiography revealed the presence of a mobile, polylobate shaped mass with mid-to-high echogenicity in the RVOT **(A)**, another lesion in the RPA **(B)**.

**Figure 2:**
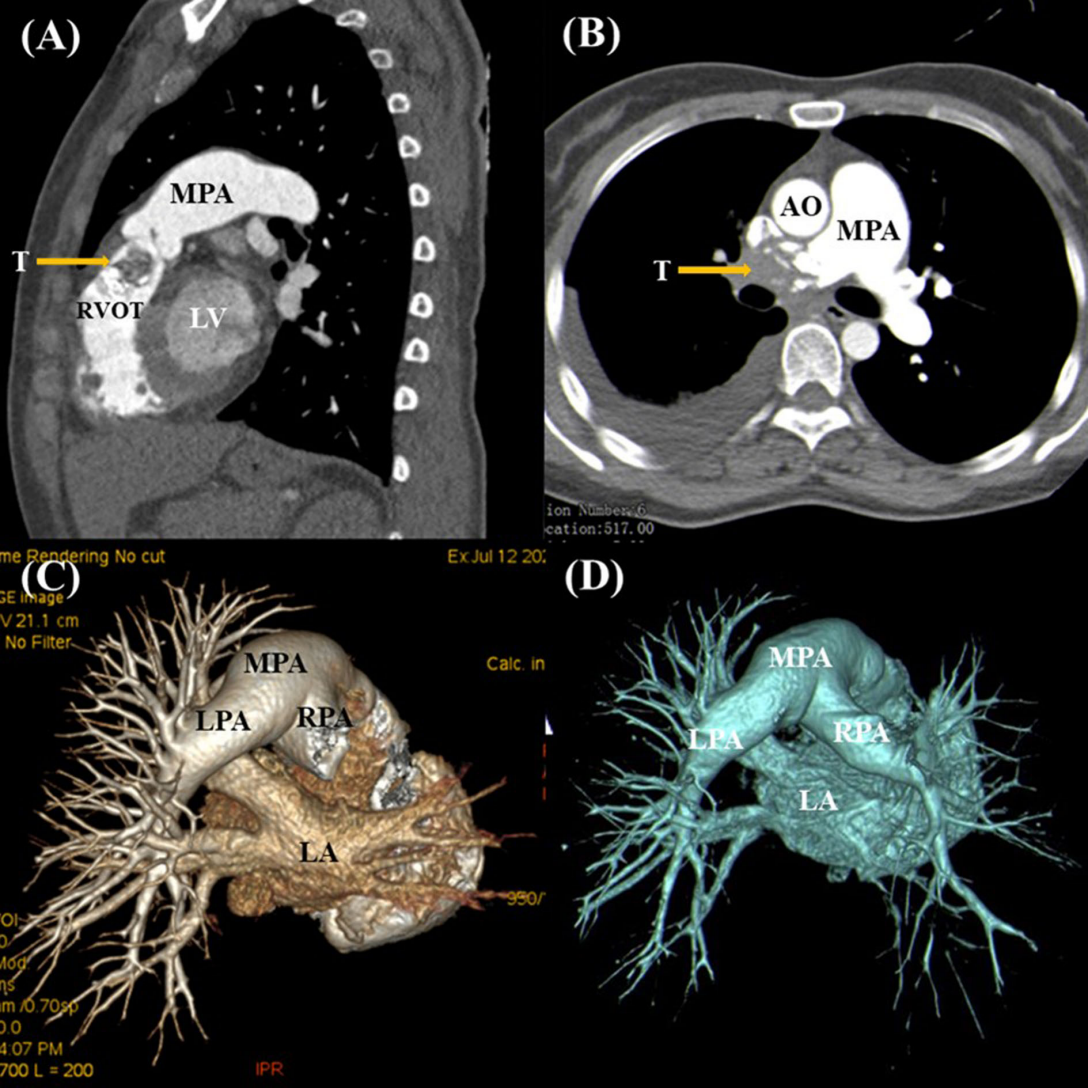
Cardiac computed tomography demonstrated a mixed-density lesion with irregular margins in the RVOT **(A)**, along with another mass in the RPA that was completely occluded **(B, C)**. Postoperative CT imaging showing re-establishment of RPA blood flow **(D)**.

**Figure 3:**
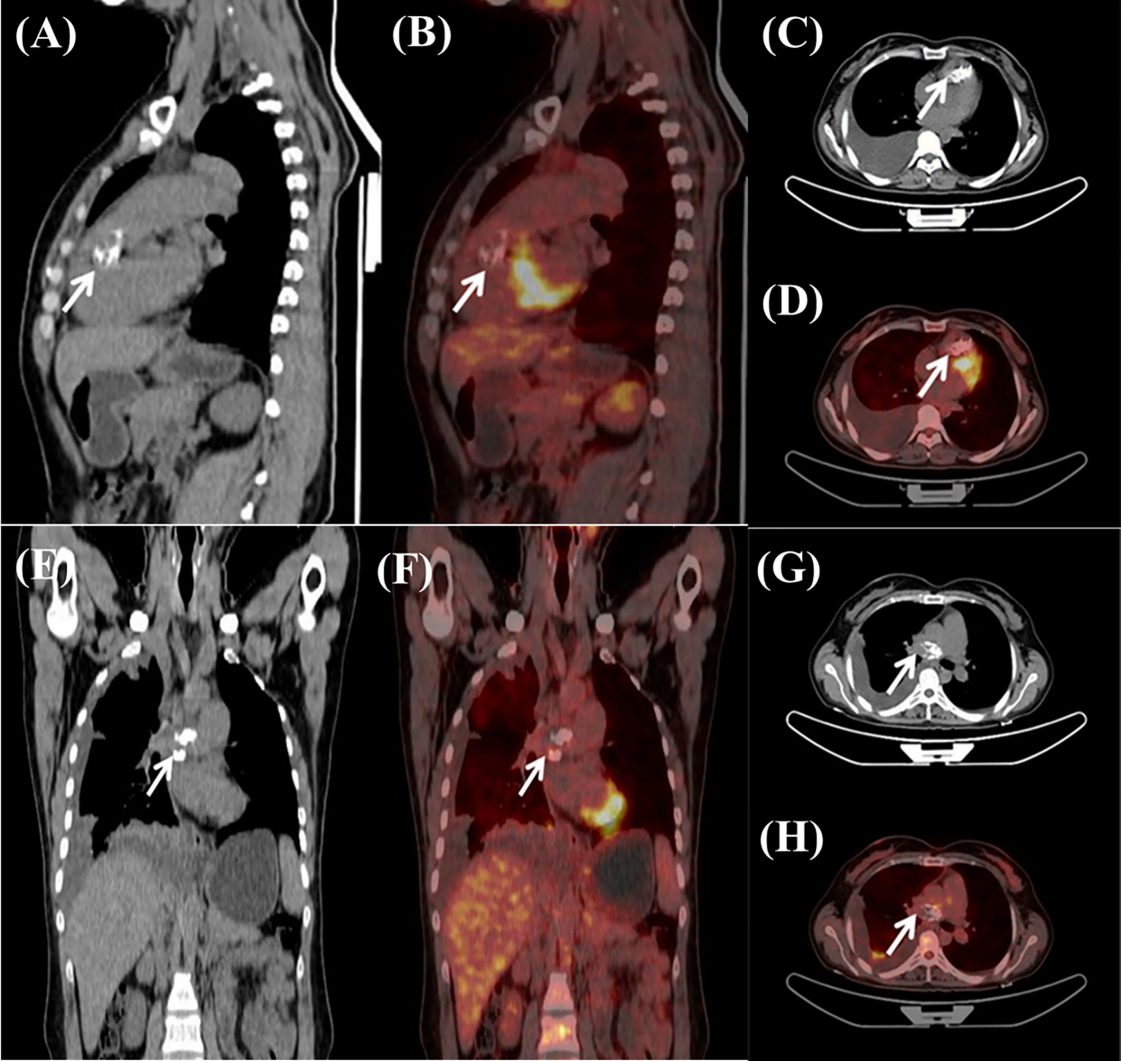
Cardiac positron emission tomography exhibited low-density mass lesions with multiple calcifications in the RVOT **(A–D)** and the RPA **(E–H)**. Notably, there were no apparent increase in glucose metabolism.

A 37-year-old female complained of chest tightness and dyspnea for a month, dry cough, and occasionally having blood streak sputum for half a month. She had no specific medical history or lifestyle. On physical examination, her heart rate was 88 beats/min, and the blood pressure was 125/74 mmHg. There is a grade II–III systolic murmur at the tricuspid valve auscultation area. The electrocardiogram showed normal sinus rhythm. Laboratory analyses were regular. Chest X-ray showed cardiomegaly and prominent proximal pulmonary arteries suggestive of pulmonary hypertension. Transthoracic echocardiography (TTE) revealed a mobile, polylobate shaped mass with mid-to-high echogenicity, measuring 36 mm × 22 mm, located in the RVOT. The demarcation between the lesion and the right ventricle wall was indistinct. Additionally, another mid-to-high echogenicity mass was observed in the RPA with occlusion. Mild tricuspid regurgitation, systolic pulmonary artery pressure of 53 mmHg, and a left ventricular ejection fraction of 60% were also noted (**Figures 1A, B**). The mass in the RVOT was considered a myxoma on TTE. Cardiac computed tomography (CT) imaging revealed the presence of a mixed-density lesion measuring 32 mm × 20 mm with irregular margins in the RVOT, leading to obstruction of blood flow to the pulmonary artery. Furthermore, a lesion was observed in the RPA, resulting in complete occlusion. Both lesions did not exhibit contrast uptake. Importantly, no pericardial effusion was detected (**Figures 2A-C**). The masses in the RVOT and RPA were distinct from each other. Additionally, a pleural effusion on the right side and inflammation in the upper lobe of the right lung were also observed. Cardiac positron emission tomography (PET) revealed the presence of low-density mass lesions in the RVOT and RPA, accompanied by multiple calcifications within these lesions. Notably, there was no observed increase in glucose metabolism (**Figure 3**). These findings suggest the potential presence of cardiac myxomas, while no indications of metastasis to the RVOT or RPA were observed during imaging.

**Figure 4:**
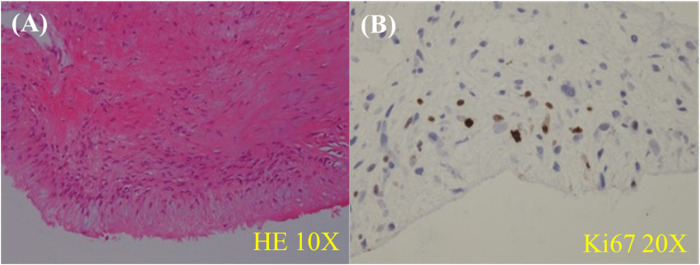
Histological examination revealed the presence of cytologic atypia in the endocardium of the right ventricle, particularly in a large core. The nuclei exhibit varying shapes, including round, oval, and spindle-shaped. Abundant fibrous hyperplasia is observed, while the number of cells is limited **(A)**. The localized lesions demonstrate spindle cell proliferation, with the presence of multinucleate myxomatous cells **(B)**. Ao, aorta; LA, left atrium; LPA, left pulmonary artery; LV, left ventricular; MPA, main pulmonary artery; ROVT, right ventricular outflow tract; RPA, right pulmonary artery; RV, right ventricle; T, tumor.

The patient underwent surgical treatment due to worsening of her clinical condition. The surgical approaches included medial sternotomy and cardiopulmonary bypass. The RVOT mass was completely excised. RPA endarterectomy was performed. A 3.5 cm tumor was removed from the intima of the RPA. Histologically, cytologic atypia was observed in the endocardium of the right ventricle, specifically in a large core. The nuclei exhibited varying shapes, including round, oval, and spindle-shaped, with no evidence of nuclear division. Fibrous hyperplasia was present in abundance, while cells were scarce. Fibrosis and calcification were observed in the pulmonary artery mass. The localized lesions demonstrated spindle cell proliferation, with multinucleate myxomatous cells also being visible. Immunohistochemistry staining revealed that the cells were negative for Calretinin, CD31, CD34, CD68, CKpan, Desmin, and S100, but positive for MDM2, 20% of cells were positive for cell proliferation index Ki-67. Upon evaluation, the right ventricular mass was diagnosed as a low-grade myxofibrosarcoma, consistent with the masses on the pulmonary arteries (**Figures 4A, B**). Thrombosis and calcification were observed in the tumors.

The patient’s postoperative progression was unremarkable, as her dyspnea and cough were alleviated following the surgical procedure. She declined any further administration of chemotherapy or radiotherapy. No discomfort was reported, and she resumed her regular activities and work. Notably, she exhibited a satisfactory recovery without any signs of recurrence during the 20-month follow-up period (**Figure 2D**).

## 3 Discussion

MFS is a malignant fibroblastic/myofibroblastic neoplasm with a prominent myxoid area (5). It is a common sarcoma subtype that occurs in the extremities and trunk of elderly patients (6). Their recurrence rates and distant metastases are high (5). Cardiac MFS is an extremely rare entity, with only a few case reports in the literature (4). Cardiac MFS arises in the endocardium and can affect any part of the heart, with LA being the most common site (7).

Histologically, they exhibit the following characteristics: a commonly nodular growth pattern; a myxoid matrix containing elongated, curvilinear capillaries; and fusiform, round, or stellate tumor cells with indistinct cell margins, slightly eosinophilic cytoplasm, and hyperchromatic atypical nuclei. The lesions ranged from hypocellular, predominantly myxoid, and predominantly spindle-cell in appearance (low-grade neoplasms) to high-grade, pleomorphic lesions (malignant fibrous histiocytoma-like) with multinucleated giant cells, high mitotic activity, and areas of necrosis (8). The lesions are classified as low-grade neoplasms with fibroblastic differentiation and lightly myxoid stroma, endocardial cellular atypia (spindle, round, and oval shaped cells), and nuclear pleomorphism. The low-grade MFS tends to metastasize locally, whereas the high-grade MFS invades local tissue and often metastasizes to distant organs such as the lung, bone, brain, and lymph system (9, 10). It was determined that the patient’s cardiac lesions were low-grade MFS.

Cardiac MFS is usually asymptomatic until an advanced stage. The clinical manifestations are variable due to a wide diversity of location, size, and morphology. They can include obstruction to flow, embolic phenomena, systemic symptoms, and local invasion causing arrhythmia or pericardial effusion (11). Left atrial MFS is solid and exhibits less infiltration than right atrial MFS, and it metastasizes at a later stage of the disease. Compared with left atrial MFS, right atrial MFS presents with larger masses and is more likely to metastasize earlier. In most cases, heart failure presents as the first symptom. In the present case, the RV mass was confined to the endocardial surface of the RVOT, which was clearly separated from the RPA mass. Many pulmonary artery sarcomas present with pulmonary artery obstruction, making them misdiagnosed as chronic pulmonary embolisms. Likewise, both clinical and imaging findings were also evident in our case.

Cardiovascular imaging remains an essential diagnostic element in the field of cardio-oncology (12). It assesses the extent of pre-existing cardiac comorbidity before making decisions regarding cancer treatment. Additionally, it serves as a benchmark for detecting alterations during therapy and for long-term follow up (13).

TTE is frequently employed as the initial diagnostic modality for the identification of cardiac tumors (14). It effectively provides information regarding the tumor’s location, size, shape, attachment, and mobility. In our particular case, TTE revealed the presence of a calcified lesion in the RVOT, as well as another space-occupying lesion in the RPA causing occlusion.

The diagnostic echocardiographic mass (DEM) score can increase cardiac masses diagnostic yield (15).The DEM score ranging from 0 to 9 was developed and validated as follows: infiltration, polylobate shape, and moderate to severe pericardial effusion were assigned 2 points each, and inhomogeneity, sessile, nonleft localization were assigned 1 point each. The patient’s DEM score was 7 points, the detailed risk score formula was as follows: infiltration (2 points), polylobate shape (2 points), inhomogeneity (1 point), sessile (1 point), and nonleft localization (1 points).

Cardiac CT scan is a critical tool for assessing cardiac tumor location, infiltration, and metastasis. It is worth noting that according to existing literature (16), irregular tumor margins, pericardial effusion, invasion, solid nature, mass diameter, CT contrast uptake, and pre-contrast characteristics are strongly indicative of the malignant nature of masses. The coexistence of at least 5 CT signs perfectly identified malignant masses. However, in this case, the CT findings did not accurately differentiate the nature of the masses.

Cardiovascular magnetic resonance (CMR) imaging is a key diagnostic tool for the evaluation of patients with suspected cardiac tumors (17). CMR provides better soft tissue characterization than CT scan in the evaluation of cardiac tumors. PET/CT is confirmed as an extremely powerful tool able to provide substantial information regarding the composition of cardiac masses (16). PET/CT scan can comprehensively assess the tumor volume, and provides functional or metabolic information. It can also reveal the aggressiveness of cancerous tumors. Based on PET/CT imaging, the patient was diagnosed with cardiac myxomas. Furthermore, there was no evidence of metastasis from elsewhere to the right heart or pulmonary artery.

Currently, surgical treatment is still the first line of treatment for primary cardiac MFS. The survival rate of patients with resectable tumors is higher than that of patients with unresectable tumors (18). These tumors often have an infiltrative growth pattern; they are difficult to excise completely, and only approximately one third of them are removed completely. During the postoperative period, patients usually develop local recurrence or metastases. Chemotherapy and radiotherapy may be considered in the case of high-grade residual lesions or tumors. Due to the limited number of cases, there is uncertainty about the role of radiotherapy and chemotherapy (9). Fortunately, our case did not show a local or distant metastasis, and the mass was resected successfully. The patient did not accept additional chemotherapy or radiotherapy for low-grade tumors. She had returned to normal activity and work. Notably, no instances of tumor recurrence or metastasis were observed during the 20-month follow-up period. Nonetheless, ongoing close monitoring remains imperative for her future care.

Primary cardiac MFS is rare malignant tumor with dismal long-term survival and limited treatment options. Surgery is the key treatment in cardiac MFS management strategy. Multiple imaging modalities are helpful for evaluating anatomical characteristics and analyzing histological characteristics of cardiac MFS. They are crucial to the patient’s prognosis and follow-up treatment.

## Data availability statement

The original contributions presented in the study are included in the article/supplementary material. Further inquiries can be directed to the corresponding author.

## Ethics statement

The studies involving humans were approved by the ethics committee of The First Affiliated Hospital of Xinjiang Medical University. The studies were conducted in accordance with the local legislation and institutional requirements. The participants provided their written informed consent to participate in this study. Written informed consent was obtained from the individual(s) for the publication of any potentially identifiable images or data included in this article.

## Author contributions

All authors contributed to design of the study. WZ and QT contributed to the data collection and drafting of the article. QT contributed to the diagnosis by echocardiography. BB performed the clinical follow-up and contributed to the manuscript. YX contributed to imaging diagnosis by CT. XS contributed to imaging diagnosis by PET/CT. QH revised the paper. All authors read and approved the final manuscript.
